# Supplementary material for: Allelic association analyses: estimation recommendations
Source: Genetics. 2026 Apr 24;233(2):iyag088. doi: 10.1093/genetics/iyag088 (PMC13232751; doi:10.1093/genetics/iyag088)
Supplement: iyag088_Supplementary_Data [file iyag088_supplementary_data.zip › Supplemental_Material_GENETICS-2026-308997.pdf]

Allelic Association Analyses: Estimation Recommendations Supplementary Information

1. Box S1: Nei's Heterozygosities.
2. Box S2: Descent Measures and Allele-sharing Statistics for Weighted Analyses.
3. Box S3: Estimators and Statistics for Cockerham approach.
4. Box S4: F_{ST} Measures of Reich et al. (2009).
5. Box S5: Descent Measures and Allele-sharing Statistics for Polyploids.
6. Figure S1: Allele sharing and standard estimates of kinship; effect of the reference population.
7. Figure S2: J. Goudet and B. Weir. A consistent estimator of kinship for admixed populations, applied to heritability studies. *TAGC2020 Online Conference* Abstract 1385B (2020).

Box S1: Nei's Heterozygosities.

Nei definitions

For population i, H_I^i is the proportion of heterozygotes and p^i is frequency of the designated allele (p^i is not the sample allele frequency $\tilde{p}^i$). The average of p^i over populations is $\check{p}$. Nei and Chesser (1983) defined three heterozygosities and three F-statistics

$$H_0 = \frac{1}{r}\sum_{i=1}^{r} H_I^i,\ \ H_S = \frac{1}{r}\sum_{i=1}^{r} H_S^i = \frac{1}{r}\sum_{i=1}^{r} 2p^i(1-p^i),\ \ H_T = 2\check{p}(1-\check{p})$$

$$F_{IS} = 1 - H_0/H_S,\ \ F_{IT} = 1 - H_0/H_T,\ \ , F_{ST} = 1 - H_S/H_T$$

Modified Estimators

Population allele and genotype frequencies are not observed, but to allow them to be affected by genetic sampling we define sample values $\tilde{H}$ of Nei's heterozygosities, shown now as allele-sharing statistics:
$\tilde{H}_0 = \frac{1}{r}\sum_{i=1}^{r}\tilde{H}_I^i = 1-\tilde{A}_I,\ \tilde{H}_W = \frac{1}{r}\sum_{i=1}^{r} 2\tilde{p}^i(1-\tilde{p}^i) = 1-\tilde{A}_W,\ \tilde{H}_T = 2\bar{p}(1-\bar{p}) = 1-\tilde{A}_T$
where $\bar{p}$ is the average sample allele frequencies $\tilde{p}^i$ over populations. We have written $\tilde{H}_W$ instead of Nei's $\tilde{H}_S$ and we add two new quantities $\tilde{H}_S = 1-\tilde{A}_S$ and $\tilde{H}_B = 1-\tilde{A}_B$. The expected values H of the $\tilde{H}$ are functions of the single allele probability π and appropriate descent measures θ to accommodate genetic sampling: $H = 2\pi(1-\pi)(1-\theta)$.

The expected values of Nei's modified F-statistics, $\tilde{F}$, where each H is replaced by $\tilde{H}$, follow from Boxes 1 and 2:

$$\mathcal{E}(\tilde{F}_{IS}) = (\theta_I - \theta_W)/(1-\theta_W),\ \ \mathcal{E}(\tilde{F}_{IT}) = (\theta_I - \theta_T)/(1-\theta_T),\ \ \mathcal{E}(\tilde{F}_{ST}) = (\theta_W - \theta_T)/(1-\theta_T)$$

From Box 1, θ_W and θ_T are explicit functions of the study dimensions n_i and r.

Unbiased Nei Estimators

In publications subsequent to Nei (1973), Nei modified his estimators to remove the explicit effect of study dimensions on their expected values. His unbiased estimator of his H_S^i in Nei and Chesser (1983) is the allele-sharing statistic $(1-\tilde{A}_S^i)$, with an average over populations of $(1-\tilde{A}_S)$, so $\hat{H}_S = \tilde{H}_S$. By "unbiased", Nei means replacing our H_W by our H_S. The average in equation (8) of Nei and Chesser (1983), however, holds only for equal sample sizes n_i and should not be used in general.

In equation (11) of Nei and Chesser (1983) there is an "unbiased" estimator $\hat{H}_T$ of total heterozygosity, derived by considering the expected value of $\tilde{H}_T$ that we know from Box 2 is $2\pi(1-\pi)(1-\theta_T)$ and can be expressed in terms of θ_B, θ_S, F_I from Box 1. Equation (11) in Nei and Chesser (1983) is an approximation that becomes exact for equal sample sizes n for $\hat{H}_T = \tilde{H}_T + (\tilde{H}_S - \tilde{H}_I/2n)/r$. From Box 2 $\tilde{H}_T = [(r-1)\tilde{H}_B + \tilde{H}_W]/r$ so $\hat{H}_T = [(r-1)\tilde{H}_B + \tilde{H}_S]/r$. This is still an explicit expression involving r.

The "unbiased" estimator F_{ST} in equation (3c) of Nei (1986) is written as $\hat{F}'_{ST} = \hat{D}'_{ST}/\hat{H}'_T$ where $\hat{H}'_T = \hat{H}_S + \hat{D}'_{ST}$. It is the same as the allele-sharing estimator if $\hat{D}'_{ST} = \tilde{H}_B - \tilde{H}_S$, $\hat{H}'_T = \tilde{H}_B$. Neither of these are explicit functions of the study dimensions.

Two substitutions make Nei's estimators in Nei (1986) the same as the allele-sharing estimators: replace $\tilde{H}_W$ by $\tilde{H}_S$ as shown in Nei and Chesser (1983), and replace $\tilde{H}_T$ by $\tilde{H}_B$ as suggested in Nei (1986). This second substitution was based on the use of a quantity D'_{ST} that has a sample value of $\tilde{H}_B$.

1. Nei M. 1973. Analysis of gene diversity in subdivided populations. Proc Natl Acad Sci. USA 70:3321-3323. doi:10.1073/pnas.70.12.3321

2. Nei M. 1977. F-statistics and analysis of gene diversity in subdivided populations. Ann Hum Genet. 41:225-233. doi:10.1111/j.1469-1809.1977.tb01918.x

3. Nei M, Chesser RK. 1983. Estimation of fixation indices and gene diversities. Ann Hum Genet. 47:253-259. doi:10.1111/j.1469-1809.1983.tb00993.x

4. Nei M. 1986. Definition and estimation of fixation indices. Evolution 40:643-645. doi:10.2307/2408586

Box S2: Descent Measures and Allele-sharing Statistics for Weighted Analyses[1].

Distinct pairs of alleles within individuals

$F_I^w = \frac{1}{n_T}\sum_{i=1}^r n_i F_I^i$

$\tilde{A}_I^w = \frac{1}{n_T}\sum_{i=1}^r n_i \tilde{A}_I^i$

$\mathcal{E}(\tilde{A}_I^w) = 1 - 2\pi(1-\pi)(1-F_I^w)$

$\mathcal{E}[\frac{1}{2}(1-\tilde{A}_I^w)] = 2\pi(1-\pi)[\frac{1}{2}(1-F_I^w)]$

Random pairs of alleles within populations

$\theta_S^w = \frac{1}{n_T}\sum_{i=1}^r n_i \theta_S^i$

$\tilde{A}_W^w = 1 - \frac{1}{n_T}\sum_{i=1}^r 2n_i\tilde{p}^i(1-\tilde{p}^i)$

$\quad = \frac{1}{n_T}\sum_{i=1}^r n_i \tilde{A}_W^i$

$\quad = \frac{1}{n_T}\sum_{i=1}^r [(n_i-1)A_S^i + \frac{1}{2}(1+A_I^i)]$

$\mathcal{E}(\tilde{A}_W^w) = 1 - 2\pi(1-\pi)\{(1-\theta_S^*) - \frac{r}{n_T}[\frac{1}{2}(1+F_I)-\theta_S]\}$

Random pairs of alleles from the whole study

$\theta_B^w = \frac{1}{(r-1)n_c n_T}\sum_{i=1}^r \sum_{i'=1 i\neq i'}^r n_i n_{i'} \theta_B^{ii'}$

$\tilde{A}_T^w = 1 - 2\bar{p}^w(1-\bar{p}^w)$

$\mathcal{E}(\tilde{A}_T^w) = 1 - 2\pi(1-\pi)\{1 - \frac{1}{n_T^2}\sum_{i=1}^r n_i^2\theta_S^i - \frac{1}{n_T^2}\sum_{i=1}^r n_i[\frac{1}{2}(1+F_I^i)-\theta_S^i] - \frac{1}{n_T}(r-1)n_c\theta_B^*\}$

$\quad = 1 - 2\pi(1-\pi)\{1 - \frac{1}{n_T^2}\sum_{i=1}^r n_i^2\theta_S^i - \frac{1}{n_T}[\frac{1}{2}(1+F_I^w)-\theta_S^w] - \frac{1}{n_T}(r-1)n_c\theta_B^w\}$

[1]: $n_T = \sum_{i=1}^r n_i, n_c = \frac{1}{r-1}\sum_{i=1}^r(n_i - \frac{n_i^2}{n_T}), \bar{p} = \frac{1}{r}\sum_{i=1}^r \tilde{p}^i, \bar{p}^w = \frac{1}{n_T}\sum_{i=1}^r n_i\tilde{p}^i$.

Box S3: Estimators and Statistics for Cockerham Approach.

WC84 Estimators[1,2]

$\hat{F}^{\rm WC}_{IS} = \frac{\rm MSI-MSG}{\rm MSI+MSG}$

$\hat{F}^{\rm WC}_{IT} = \frac{(\rm MSP-MSG)+n_c(\rm MSI-MSG)}{(\rm MSP-MSI)+n_c(\rm MSI+MSG)}$

$\hat{F}^{\rm WC}_{ST} = \frac{\rm MSP-MSI}{(\rm MSP-MSI)+n_c(\rm MSI+MSG)}$

Between populations.

WC84 notation: $\mathrm{SSP} = 2n_T\tilde{p}^w(1-\tilde{p}^w) - \sum_{i=1}^r 2n_i\tilde{p}^i(1-\tilde{p}^i)$

WG17 notation: $\mathrm{SSP} = n_T[(1-\tilde{A}^w_T) - (1-\tilde{A}^w_W)]$

$\mathrm{MSP} = \frac{1}{r-1}\mathrm{SSP}$

WC84 model: $\mathcal{E}(\mathrm{MSP}) = 2\pi(1-\pi)[\frac{1}{2}(1-F_I) + (F_I - \theta_S) + n_c(\theta_S - \theta_B)]$

WG17 model: $\mathcal{E}(\mathrm{MSP}) = 2\pi(1-\pi)\frac{1}{r-1}\sum_{i=1}^r\{(1-\frac{n_i}{n_T})[\frac{1}{2}(1-F_I^i) + (F_I^i - \theta_S^i) + n_{i_c}(\theta_S^i - \theta_B^w)]\}$

Between individuals within populations.

WC84 notation: $\mathrm{SSI} = \sum_{i=1}^r 2n_i\tilde{p}^i(1-\tilde{p}^i) - \frac{1}{2}\sum_{i=1}^r n_i\tilde{H}_I^i$

WG17 notation: $\mathrm{SSI} = n_T[(1-\tilde{A}^w_W) - \frac{1}{2}(1-\tilde{A}^w_I)]$

$\mathrm{MSI} = \frac{1}{n_T-r}\mathrm{SSI}$

WC84 model: $\mathcal{E}(\mathrm{MSI}) = 2\pi(1-\pi)[\frac{1}{2}(1-F_I) + (F_I - \theta_S)]$

WG17 model: $\mathcal{E}(\mathrm{MSI}) = 2\pi(1-\pi)\frac{1}{n_T-r}\sum_{i=1}^r(n_i-1)[\frac{1}{2}(1-F_I^i) + (F_I^i - \theta_S^i)]$

Within Individuals.

WC84 notation: $\mathrm{SSG} = \frac{1}{2}\sum_{i=1}^r n_i\tilde{H}_I^i$

WG17 notation: $\mathrm{SSG} = \frac{1}{2}n_T(1-\tilde{A}^w_I)$

$\mathrm{MSG} = \frac{1}{n_T}\mathrm{SSG}$

WC84 model: $\mathcal{E}(\mathrm{MSG}) = 2\pi(1-\pi)[\frac{1}{2}(1-F_I)]$

WG17 model: $\mathcal{E}(\mathrm{MSG}) = 2\pi(1-\pi)\frac{1}{2}(1-F_I^w) = 2\pi(1-\pi)\frac{1}{n_T}\sum_{i=1}^r n_i\frac{1}{2}(1-F_I^i)$

[1]: WC84 model: $F_I^i = F_I, \theta_S^i = \theta_S$ for all i, and $\theta_B^w = \theta_B$.

[2]: $n_T = \sum_{i=1}^r n_i, n_{i_c} = (n_i - \frac{n_i^2}{n_T}), n_c = \frac{1}{r-1}\sum_{i=1}^r n_{i_c}, \bar{p} = \frac{1}{r}\sum_{i=1}^r\tilde{p}^i, \bar{p}^w = \frac{1}{n_T}\sum_{i=1}^r n_i\tilde{p}^i$.

WC84: Weir BS, Cockerham CC. 1984. Estimating F-statistics for the analysis of population structure. Evolution 38:1358-1370. doi.org/10.1111/j.1558-5646.1984.tb05657.x.

WG17: Weir BS, Goudet J. 2017. A unified characterization of population structure and relatedness. Genetics 206:2085-2103. doi.org/10.1534/genetics.116.198424

Box S4: f-statistics of Reich *et al.* (2009).

Reich *et al.* (2009) treat inferences about population-pair coancestry terms $\theta_S^{ii'}$ for populations i, i'. If $\tilde{p}^i$ is the designated allele frequency in a sample from population i, the allele-sharing statistics are:
$\tilde{A}_W^i = (\tilde{p}^i)^2 + (1-\tilde{p}^i)^2$, $\tilde{A}_B^{ii'} = \tilde{p}^i\tilde{p}^{i'} + (1-\tilde{p}^i)(1-\tilde{p}^{i'})$
and the numerators of sample f-statistics are:
$\mathcal{N}_2(i,i') = (\tilde{p}^i - \tilde{p}^{i'})^2 = \frac{1}{2}(\tilde{A}_W^i + \tilde{A}_W^{i'}) - \tilde{A}_B^{ii'}$
$\mathcal{N}_3(i:i',i'') = (\tilde{p}^i - \tilde{p}^{i'})(\tilde{p}^i - \tilde{p}^{i''}) = \frac{1}{2}(\tilde{A}_W^i - \tilde{A}_B^{ii'} - \tilde{A}_B^{ii''} + \tilde{A}_B^{i'i''})$
$\mathcal{N}_4(i,i':i'',i''') = (\tilde{p}^i - \tilde{p}^{i'})(\tilde{p}^{i''} - \tilde{p}^{i'''}) = \frac{1}{2}(\tilde{A}_B^{ii''} + \tilde{A}_B^{i'i'''} - \tilde{A}_B^{ii'''} - \tilde{A}_B^{i'i''})$
With a common denominator of $\mathcal{D} = (1-\tilde{A}_B)$ where $\tilde{A}_B = \sum_{i=1}^r \sum_{i'=1 i\neq i'}^r \tilde{A}_B^{ii'}/[r(r-1)]$,
the sample f statistics are $\tilde{f}_x = \mathcal{N}_x/\mathcal{D}$ for $x = 2,3,4$ and these have expected values,
$\mathcal{E}[\tilde{f}_2(i,i')] = [(\theta_W^i + \theta_W^{i'})/2 - \theta_B^{ii'}]/(1-\theta_B)$ where $\theta_W^i = \theta_S^i + [(1+F_I^i)/2 - \theta_S^i]/n_i$
$\mathcal{E}[\tilde{f}_3(i:i',i'')] = \theta_W^i - \theta_B^{ii'} - \theta_B^{ii''} + \theta_B^{i'i''}$
$\mathcal{E}[\tilde{f}_4(i,i':i'',i''')] = \theta_B^{ii''} + \theta_B^{i'i'''} - \theta_B^{ii'''} - \theta_B^{i'i''}$

To reduce the bias in these estimators, Reich *et al.* subtract $[\tilde{p}^i(1-\tilde{p}^i)/(2n_i-1) + \tilde{p}^{i'}(1-\tilde{p}^{i'})/(2n_{i'}-1)]$ from $\mathcal{N}_2(i,i')$, in effect replacing $\tilde{A}_W^i$ by $\tilde{A}_D^i$ and θ_W^i by $\theta_D^i = \theta_S^i + (F_I^i - \theta_S^i)/(2n_i-1)$.

To remove the effects of Hardy-Weinberg disequilibrium, i.e. $F_I^i \neq \theta_S^i$, Reich *et al.* took account of individuals being heterozygous by replacing $\tilde{A}_W^i$ by $\tilde{A}_S^i$ in $\mathcal{N}_2(i,i')$:
$\mathcal{N}_2(i,i') = [\frac{1}{2}(\tilde{A}_W^i + \tilde{A}_W^{i'}) - \tilde{A}_B^{ii'}] + \frac{1}{4n_i}(1-\tilde{A}_I^i) + \frac{1}{4n_{i'}}(1-\tilde{A}_I^{i'}) = [\frac{1}{2}(\tilde{A}_S^i + \tilde{A}_S^i) - \tilde{A}_B^{ii'}]$
The expected values of the modified f-statistics estimators are
$\mathcal{E}[\hat{f}_2(i,i')] = \frac{1}{2}(k_{SB}^i + k_{SB}^{i'}) - k_{SB}^{ii'}$
$\mathcal{E}[\hat{f}_3(i:i',i'')] = \frac{1}{2}(k_{SB}^i - k_{SB}^{ii'} - k_{SB}^{ii''} + k_{SB}^{i'i''})$
$\mathcal{E}[\hat{f}_4(i,i':i'',i''')] = \frac{1}{2}(k_{SB}^{ii''} + k_{SB}^{i'i'''} - k_{SB}^{ii'''} - k_{SB}^{i'i''})$
where $k_S^i = (\theta_S^i - \theta_B)/(1-\theta_B) = F_{ST}^i$ and $k_B^{ii'} = (\theta_B^{ii'} - \theta_B)/(1-\theta_B)$.

Patterson N, Moorjani F, Luo Y, Mallik S, Rohland N, Zhan YP, Genschorek T, Webster T, Reich D. 2012. Ancient admixture in human history. Genetics 192:1065-1093. doi.org/10.1534/genetics.112.145037

Reich A, Thangaraj K, Patterson N, Price AL, Singh L. 2009. Reconstructing Indian population history. Nature 461:489-494. doi.org/10.1038/nature08365

Box S5: Descent Measures and Allele-sharing Statistics for Polyploids[1].

Distinct pairs of alleles within individuals.

$\tilde{A}^i_j = \dfrac{1}{\kappa(\kappa-1)}[X^i_j(X^i_j - 1) + (\kappa - X^i_j)(\kappa - X^i_j - 1)]$

$\mathcal{E}(\tilde{A}^i_j) = 1 - 2\pi(1-\pi)(1 - F^i_j)$

Pairs of alleles, one from two distinct individuals.

$\tilde{A}^i_{jj'} = \frac{1}{\kappa^2}[X^i_j X^i_{j'} + (\kappa - X^i_j)(\kappa - X^i_{j'})] = \frac{1}{2\kappa^2}[\kappa^2 + (2X^i_j - \kappa)(2X^i_{j'} - \kappa)]$

$\mathcal{E}(\tilde{A}^i_{jj'}) = 1 - 2\pi(1-\pi)(1 - \theta^i_{jj'})$

$\tilde{A}^i_S = \frac{1}{n_i(n_i-1)} \sum_{j=1}^{n_i} \sum_{j'=1 j\neq j}^{n_i} \tilde{A}^i_{jj'}$

$\mathcal{E}(\tilde{A}^i_S) = 1 - 2\pi(1-\pi)(1 - \theta^i_S)$

Estimators for individuals and pairs of individuals

$\hat{f}^i_j = \frac{\tilde{A}^i_j - \tilde{A}^i_S}{1 - \tilde{A}^i_S}$

$\mathcal{E}(\hat{f}^i_j) = \frac{F^i_j - \theta^i_S}{1 - \theta^i_S}$

$\hat{k}^i_{jj'} = \frac{\tilde{A}^i_{jj'} - \tilde{A}^i_S}{1 - \tilde{A}^i_S}$

$\mathcal{E}(\hat{k}^i_{jj'}) = \frac{\theta^i_{jj'} - \theta^i_S}{1 - \theta^i_S}$

[1] κ is ploidy level.

Figure S1: Allele sharing and standard estimates of kinship; effect of the reference population

We used the African populations from the 1000 genomes (Bryska-Bishop *et al.* 2022) to illustrate the perfect concordance of allele-sharing estimates whatever the reference set. On panel a of Figure S1 we compare the allele Sharing estimates $\hat{\boldsymbol{K}}_{AS}$ using either the continent (X-axis) or the whole world (i.e. study) (Y-axis) as a reference and we see the expected perfect concordance between estimates based on the continent or the whole world reference. We note estimates with the whole world as a reference are lower than those with the continent as a reference, and this is expected, as African genomes are more heterozygous and therefore differ more among themselves than a random pair of genomes from the world. Panel b shows the standard estimates $\hat{\boldsymbol{K}}_{Std}$ for the same set of individuals, with either continent (X-axis) or world (Y-axis) as a reference. $\hat{\boldsymbol{K}}_{Std}$ for pairs of individuals from the ASW population are shown in red. We see that for this population in particular, rankings of $\hat{\boldsymbol{K}}_{Std}$ can be opposite depending of the reference set: pairs of ASW individuals have a standard kinship close to 0 when the reference is the continent, but standard kinship of 0.125 when the reference is the world. Yet other pairs have a standard kinship of 0.12 when the reference is the continent but a slightly negative standard kinship when the reference is the world.

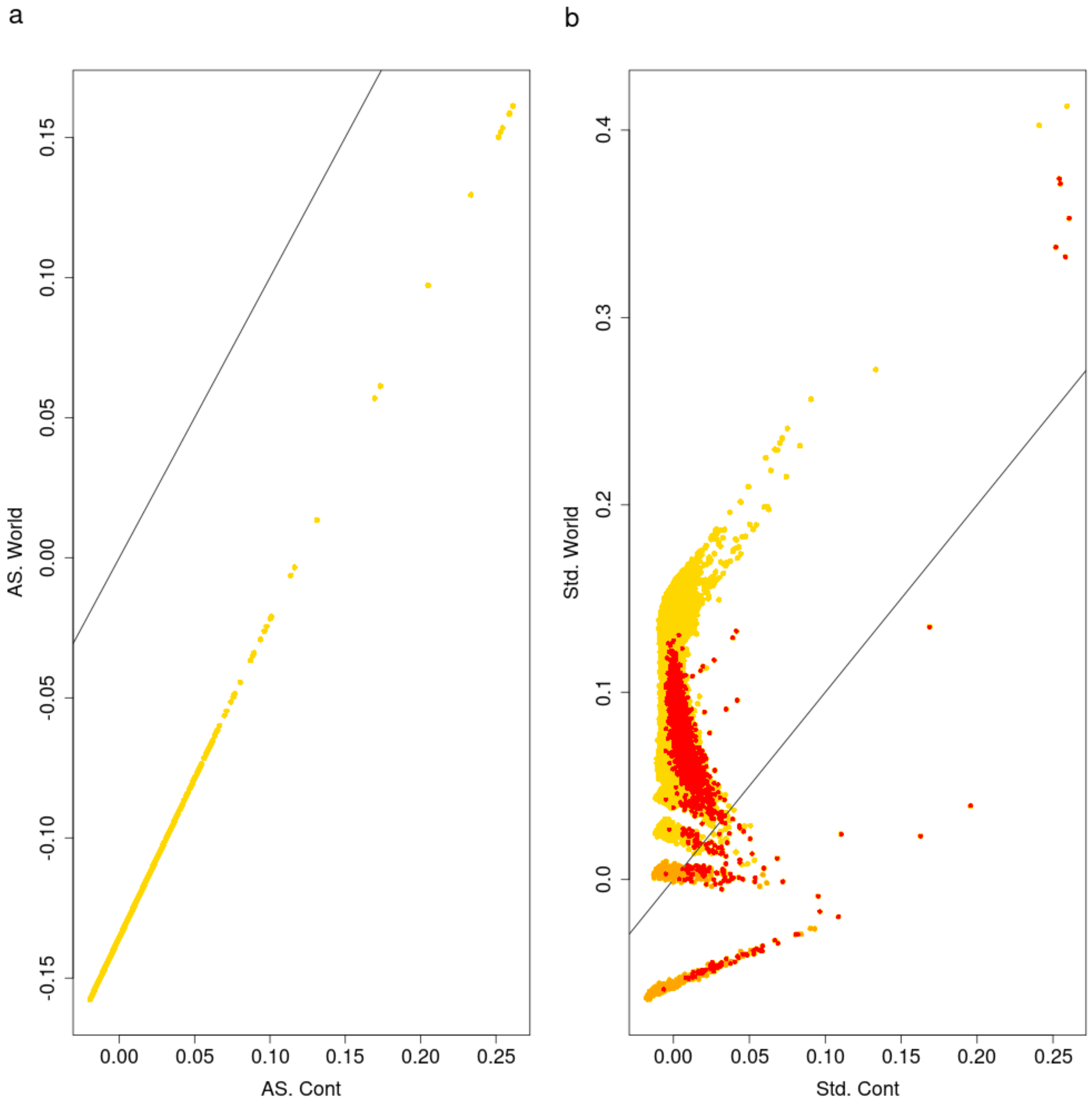

Figure S1: a. Allele sharing individual-pair kinship estimates $\hat{\mathbf{K}}_{AS}$ for African samples, using either the continent or the world as a reference. b. Standard individual-pair kinship estimates $\hat{\mathbf{K}}_{Std}$ for the same African samples, using either the continent or the world as a reference. In red are pairs of samples from a population of African Ancestry in South West USA (population ASW).

Figure S2: Goudet and Weir (2021)

.

Unil | Université de Lausanne
Département d'écologie et évolution

DEPARTMENT OF BIOSTATISTICS
SCHOOL OF PUBLIC HEALTH
UNIVERSITY *of* WASHINGTON

A consistent estimator of kinship for admixed populations, applied to heritability estimation.

Jérôme Goudet (jerome.goudet@unil.ch) and Bruce Weir (bsweir@uw.edu)

Quantitative Trait Variance

For GRM $\boldsymbol{G}$ with elements $(1+F_i)$ on the diagonal, and elements $2\theta_{ij}$ off the diagonal, the $n\times 1$ vector $\boldsymbol{Y}$ of trait values has variance

$$\mathrm{Var}(\boldsymbol{Y}) = \boldsymbol{G}\sigma_A^2 + \boldsymbol{I}\sigma_e^2$$

The trace of this matrix is

$$\mathrm{tr}(\boldsymbol{G}) = \sum_{i=1}^{n} G_{ii} = n(1+F_W)$$

to define the average inbreeding F_W for the sample, and the sum of the off-diagonal elements is

$$\Sigma_{\boldsymbol{G}} \quad = \quad \sum_{i=1}^{n}\sum_{\substack{j=1\\ i\neq j}}^{n} G_{ij} = 2n(n-1)\theta_S$$

to define the average kinship θ_S for the sample.

Speed et al.

Speed et al. calculated two variances, $\hat{V}_Y$ for the sample variance of trait values and $\hat{V}_R$ for the residual variance once the genotypic effects have been fitted, to estimate heritability:

$$\widehat{h^2} = \frac{\hat{V}_Y - \hat{V}_R}{\hat{V}_Y}$$

If F_W, θ_S are known, we find that

$$\mathcal{E}(\widehat{h^2}) \quad = \quad \frac{(1+F_W-2\theta_S)\sigma_A^2}{(1+F_W-2\theta_S)\sigma_A^2+\sigma_e^2}$$

to emphasize the role of both inbreeding and kinship.

Use of Allele-sharing GRM

Weir & Goudet, 2017, estimate half the GRM by $\boldsymbol{K}_{as}$:

$$\boldsymbol{K}_{\mathrm{as}_{\mathrm{ij}}} = \frac{\tilde{M}_{ij} - \tilde{M}_S}{1-\tilde{M}_S}$$

$\tilde{M}_{ij}$ is allele-sharing for individuals i, j, with mean $\tilde{M}_S$ over $i \neq j$. In this case $\Sigma_{\boldsymbol{K}_{\mathrm{as}}}$ is 0 and $\mathcal{E}[\mathrm{tr}(\boldsymbol{K}_{as})]$ is $n(1+f_W)/2$, where $f_W = (F_W-\theta_S)/(1-\theta_S)$ is the within-population inbreeding coefficient (i.e. F_{IS}). Therefore

$$\mathcal{E}(\widehat{h}^2) = \frac{(1+f_W)\sigma_A^2}{(1+f_W)\sigma_A^2+\sigma_e^2}$$

This replaces F_W (i.e. F_{IT}) in the classical result with f_W, reflecting that it is f_W and not F_W that can be estimated with data from a single population.

Use of Standard GRM

When half the GRM is estimated by $\boldsymbol{K}_{\mathrm{Std}}$ with elements

$$\frac{\sum_l (X_{il}-2\tilde{p}_l)(X_{jl}-2\tilde{p}_l)}{\sum_l 4\tilde{p}_l(1-\tilde{p}_l)}$$

we find for large n, the same estimate of heritability, even though $\boldsymbol{K}_{\mathrm{Std}} \neq \boldsymbol{K}_{\mathrm{as}}$.

GCTA (Yang et al, 2011) modifies the diagonal elements of the GRM to

$$\frac{1}{L}\sum_{l=1}^{L}\frac{[X_{il}^2 - 2(1+\tilde{p}_l)X_{il} + 2\tilde{p}_l]}{4\tilde{p}_l(1-\tilde{p}_l)}$$

If the average over SNPs of ratios is changed to the ratio of averages, this matrix also gives the same heritability estimate as does $\boldsymbol{K}_{\mathrm{as}}$.

Relationship Between GRMs

For large sample sizes, the standard matrix is the double-centered allele-sharing one:

$$\boldsymbol{K}_{\mathrm{Std}} = (\boldsymbol{I} - \frac{1}{n}\boldsymbol{J})\boldsymbol{K}_{\mathrm{as}}(\boldsymbol{I} - \frac{1}{n}\boldsymbol{J})$$

where $\boldsymbol{J}$ is an $n\times n$ matrix with every element equal to 1.

We also find that, for large n, $\mathrm{tr}(\boldsymbol{K}_{\mathrm{as}})$, $\mathrm{tr}(\boldsymbol{K}_{\mathrm{Std}})$, $\mathrm{tr}(\boldsymbol{K}_{\mathrm{GCTA}})$ are all the same, but only $\boldsymbol{K}_{\mathrm{as}}$ gives inbreeding and kinship estimates that rank individuals consistently across different reference sample sets.

Numerical Results

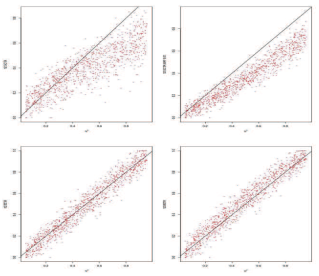

Results for 1,000 simulated traits using 1000 Genomes data. 10,000 causal loci were drawn randomly from chrs 1 and 2. X axis shows true heritabilities, Y axis shows estimates,

Top row uses $\boldsymbol{K}_{\mathrm{GCTA}}$ with average of ratios for combining SNPs. Bottom row uses $\boldsymbol{K}_{\mathrm{as}}$ as shown above.

Left column uses all SNPs, right column uses only SNPs with MAF$>$ 0.01.

References

Speed et al. 2012 AJHG 91:1011
Weir, Goudet. 2017 Genetics 206:2085
Yang et al. 2011 AJHG 88:76

Support

Swiss NSF 31003A-138180,IZKOZ3-157867, US NIH GM075091.
